# Direct Cardiac Reprogramming: Advances in Cardiac Regeneration

**DOI:** 10.1155/2015/580406

**Published:** 2015-06-15

**Authors:** Olivia Chen, Li Qian

**Affiliations:** Department of Pathology and Laboratory Medicine, McAllister Heart Institute, University of North Carolina, Chapel Hill, NC 27599, USA

## Abstract

Heart disease is one of the lead causes of death worldwide. Many forms of heart disease, including myocardial infarction and pressure-loading cardiomyopathies, result in irreversible cardiomyocyte death. Activated fibroblasts respond to cardiac injury by forming scar tissue, but ultimately this response fails to restore cardiac function. Unfortunately, the human heart has little regenerative ability and long-term outcomes following acute coronary events often include chronic and end-stage heart failure. Building upon years of research aimed at restoring functional cardiomyocytes, recent advances have been made in the direct reprogramming of fibroblasts toward a cardiomyocyte cell fate both *in vitro* and *in vivo*. Several experiments show functional improvements in mouse models of myocardial infarction following *in situ* generation of cardiomyocyte-like cells from endogenous fibroblasts. Though many of these studies are in an early stage, this nascent technology holds promise for future applications in regenerative medicine. In this review, we discuss the history, progress, methods, challenges, and future directions of direct cardiac reprogramming.

## 1. Introduction

**Figure 1:**
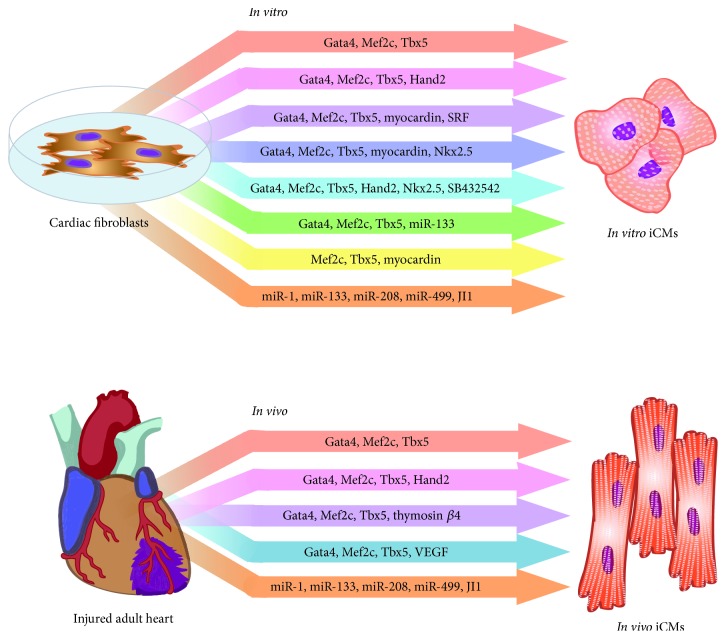
Methods of direct cardiac reprogramming. Several approaches for converting mouse or rat fibroblasts to cardiomyocyte-like cells* in vitro* and* in vivo* have been reported and are summarized here.* In vitro* reprogramming predominantly yields partially reprogrammed cells, while* in vivo* reprogramming yields more mature, fully reprogrammed cardiomyocyte-like cells. iCMs denotes induced cardiomyocytes.

Cardiac diseases cause major morbidity and mortality worldwide. The American Heart Association's yearly review of cardiovascular disease reports that 1 out of every 7 deaths in the United States in 2011 was caused by coronary heart disease [1]. Coronary heart disease predisposes to acute cardiac injuries such as myocardial infarction (MI), which often result in significant and permanent losses of functional cardiomyocytes. Because the human heart has minimal ability for endogenous cardiomyocyte regeneration [2], reparative responses to cardiac cell death primarily rely on activated fibroblasts forming scar tissue. This response to injury fails to restore cardiomyocyte function and often leads to undesirable outcomes including development of arrhythmias and chronic heart failure. Unfortunately, short of heart transplantation, current therapies do not restore declining pump function following cardiac injury. Efforts to address this issue have led to the exploration of several cellular approaches aimed at restoring functional cardiomyocytes; these include stimulating expansion and differentiation of endogenous cardiac progenitor cells and lineage differentiation of embryonic stem cells or induced pluripotent stem cells. An alternative approach centers on converting nonmyocyte cells directly into new cardiomyocytes (Figure 1). Recently, promising advances have been achieved by directly reprogramming mouse cardiac fibroblasts (CFs) into functional cardiomyocytes* in vitro* and* in vivo* by transcription factor expression [3, 4]. Specific combinations of microRNA (miRs) have also been reported to yield similar reprogramming [5]. In human cells, direct reprogramming has also been used to convert dermal and cardiac fibroblasts to cardiomyocytes by specific transcription factor combinations or combinations of transcription factors and miRs [6–8]. Together, these studies describe exciting developments in direct cardiac reprogramming and highlight the potential that cellular therapies hold for future regenerative cardiac therapies. Here, we review the history, recent advances, major challenges, and future directions of direct cardiac reprogramming.

## 2. Master Genes Regulate Cell Fate

In 1986, the Weintraub laboratory [9] laid the groundwork for muscle cell reprogramming by demonstrating that somatic cells could be induced to adopt a skeletal muscle cell fate* in vitro*, through overexpression of the skeletal muscle master regulator gene,* MyoD*. Their experiments showed that forced expression of the* MyoD* gene, which encodes a transcription factor containing a basic helix-loop-helix (bHLH) domain, was capable of inducing expression of several muscle-specific proteins including myosin heavy chain and muscle creatinine kinase [10]. The ability of master regulator genes to govern cell fate changes* in vivo* was later shown by Halder et al. [11] who ectopically expressed* eyeless*, the fly homologue of the vertebrate* Pax6* gene, in* Drosophila* and observed ectopic formation of eye structures. Similarly, Santerre et al. [12] showed that transgenic mice overexpressing* bmyf*, the bovine homolog of the human bHLH domain-containing, myogenic determination transcription factor gene* myf5*, developed skeletal-muscle like cells in the brain. Together, these studies led to the recognition that* in vivo *ectopic expression of specific master regulator genes could stimulate cell fate changes. Indeed,* in vivo* reprogramming was also achieved by Murry et al. [13] who reported that adenovirus delivery of* MyoD* to cryoinjured myocardium in rats could cause cardiac fibroblast conversion to skeletal muscle cells. However, successful myogenic conversion, determined by coexpression of* MyoD* and sarcomere myosin, required high viral doses and occurred in only 14% of* MyoD*-expressing cells. The lower efficiency of conversion suggests the existence of substantial barriers to the described method of skeletal muscle reprogramming. Nevertheless, these discoveries formed the foundation of direct cellular reprogramming by transcription factor expression and served as an impetus for current advancements in the generation of induced cardiomyocytes.

## 3. Induced Pluripotency and Direct Cellular Reprogramming

In the 1960s, Gurdon [14] challenged the view of permanently fixed cell fates by showing that transfer of a somatic cell nucleus into an enucleated frog egg could reprogram the somatic nucleus to a pluripotent state. In 2006, Takahashi and Yamanaka [15] discovered that conversion of somatic cells to pluripotency could be achieved by coexpressing four transcription factors. They successfully reprogrammed mouse fibroblasts to induced pluripotent stem cells (iPSCs) using transcription factors Oct4, Sox2, Klf4, and c-Myc (OSKM). The possibility of changing somatic cells to potentially any other cell lineages sparked many exciting developments in iPSC technology, which are summarized in several other reviews [16, 17]. Advances in iPSC technology also encouraged novel directions in direct cellular reprogramming. Breaking from the* MyoD* model of single transcription factor-mediated reprogramming, laboratories worldwide began reporting combinations of transcription factors that could induce different cell fate changes. Efe et al. [18] reported that addition of a small molecule Janus kinase (JAK) inhibitor in mouse embryonic fibroblasts (MEFs) transiently expressing OSKM factors in reprogramming media modified to hinder iPSC induction and promote cardiomyogenesis resulted in formation of spontaneously contracting cardiomyocyte colonies. While there was a minute (1%) population of iPSC-like cells achieved following this method, the authors determined that iPSC and cardiomyocyte generation were parallel processes; that is, reprogramming to cardiac cell fate was unlikely to have passed through a pluripotent intermediate [18]. This study was the first to show cardiomyocyte generation from MEFs by the combination of transcription factor expression, specific culture conditions, and small molecules. The breadth of direct reprogramming applications for generating a variety of cell lineages has also been demonstrated by many other laboratories. Utilizing a specific combination of three transcription factors, Zhou et al. [19] directly reprogrammed exocrine pancreas cells into insulin-secreting pancreatic *β*-cells* in vivo*. Shortly thereafter, direct reprogramming of fibroblasts into other cell lineages generated neurons [20], hepatocytes [21], endothelial cells [22], hematopoietic stem cells [23], and cardiomyocytes [4]. Herein, we will discuss advances specifically related to direct cardiac reprogramming.

## 4. *In Vitro* Direct Cardiac Reprogramming

With the success of reprogramming to pluripotency achieved through expression of a combination of transcription factors, efforts to identify a master regulator for cardiomyocyte transdifferentiation shifted toward efforts to identify multiple factors that together could activate the cardiac program. In 2010, Ieda et al. [4] identified a combination of three transcription factors, Gata4, Mef2c, and Tbx5 (GMT), that was sufficient to convert mouse cardiac and dermal fibroblasts into functional cardiomyocyte-like cells* in vitro*. In order to identify the GMT reprogramming factors, the authors created an* in vitro* assay to determine the minimal number of factors required to convert nonmyocyte cells into a cardiomyocyte cell fate. Transgenic mice were generated to express enhanced green fluorescent protein (EGFP) driven by the *α-*myosin heavy chain (*α*MHC) promoter, which is activated in mature cardiomyocytes and thus permits their identification by green fluorescence. Cardiac fibroblasts were extracted from neonatal transgenic mice and *α*MHC-EGFP^−^/Thy1^**+**^ fibroblasts were purified by fluorescence-activated cell sorting (FACS). Fourteen transcription factors of known importance in cardiac development were then retrovirally overexpressed in the purified cardiac fibroblasts. A small number of EGFP-positive cells were observed, suggesting successful cell fate conversion. Serial deletion of individual transcription factors from the original pool of 14 transcription factors yielded the three transcription factors, GMT, which together were capable of inducing EGFP expression in 15–20% of cells, termed induced cardiomyocytes (iCMs). While some iCMs expressed additional cardiac markers like cardiac Troponin T (cTnT) and sarcomeric *α*-actinin, assembled sarcomere structures, and demonstrated global enrichment in transcripts similar to the cardiomyocyte transcriptome, the majority of iCMs were likely only partially reprogrammed.

Despite this, many iCMs could generate Ca^2+^ transients and 4 weeks after reprogramming, spontaneous beating was observed in ≈0.5% of *α*MHC-EGFP^**+**^/cTnT^**+**^ iCMs, which also exhibited action potentials comparable to those of adult ventricular cardiomyocytes [4]. Furthermore, iCMs demonstrated epigenetic shifts toward a neonatal cardiomyocyte-like state at the cardiac-specific genes Actn2, Ryr2, and TnnT2, suggesting that reprogramming with the GMT factors induces changes in the epigenetic status of reprogramming cells. While it is possible that the observed iCMs were actually remote cardiac progenitor cells contaminating the fibroblast population, this is unlikely for two reasons. First, use of genetic lineage tracing showed that iCMs did not express cardiac progenitor markers (i.e., Mesp1 and Isl1) during reprogramming. Second, the GMT factors also successfully reprogrammed 15% of neonatal tail-tip fibroblasts (TTF) to *α*MHC-EGFP^**+**^ iCMs, which also showed expression of cTnT and generation of Ca^2+^ transients; these TTF-derived iCMs, however, did not beat and the efficiency of their generation was reduced. While these results demonstrate exciting developments in direct cardiac reprogramming, the efficiency of* in vitro* direct reprogramming by this method is low (<1%) but comparable to the efficiency of iPSC generation [4, 15]. Shortly after this study, Song et al. [24] discovered that addition of Hand2, a bHLH transcription factor, to the GMT cocktail of factors (together termed GHMT) could induce adult TTFs into beating iCMs. In identifying Hand2, Song et al. [24] isolated cardiac fibroblasts from *α*MHC-GFP transgenic mice and retrovirally expressed six core transcription factors involved in cardiac development: Hand2, Mesp1, Nkx2.5, and the GMT factors. Serial deletion of transcription factors revealed several factor combinations that efficiently induced GFP expression. In examining these factors for their ability to induce cTnT expression in adult TTFs, the GHMT cocktail was identified as producing a 4-fold increased conversion of adult TTFs to *α*MHC-EGFP^**+**^/cTnT^**+**^ iCMs compared to GMT reprogramming of neonatal TTFs. These TTF-derived iCMs formed sarcomeres, generated Ca^2+^ transients, and showed a cardiomyocyte-like transcriptome profile. Additionally, 5 weeks after GHMT reprogramming of TTFs, a small population of these iCMs were observed to beat spontaneously, demonstrating that GHMT could directly reprogram TTFs to functional iCMs. In a different study with the GHMT factors, Hirai et al. [25] fused the GHMT factors to the* MyoD* activation domain and observed accelerated reprogramming and increased numbers of beating iCMs. More recently, Nam et al. [26] demonstrated that* in vitro *GHMT reprogramming of fibroblasts into cardiomyocytes resulted in a range of phenotypes, including immature forms of atrial, ventricular, and pacemaker cells. Using multiplex immunostaining and patch clamping, the authors first identified unique constellations of cardiac markers and electrical activity that reliably defined atrial, ventricular, and pacemaker cardiomyocyte subtypes in Hcn4-GFP reporter mice, where GFP expression is driven by the promoter of Hcn4, a pacemaker-specific potassium channel gene. On a single cell level, they determined that GHMT reprogramming of MEFs derived from Hcn4-GFP reporter mice resulted in iCMs with immature phenotypes of all three major forms of cardiac cells, thus demonstrating an exceptional degree of plasticity intrinsic to GHMT reprogramming and providing a starting point for deciphering cardiac cell subtype-specific reprogramming.

The previously described methods by Ieda et al. [4] and Song et al. [24] for identifying reprogramming factors relies on *α*MHC-EGFP reporter activation. Although this may hone in on crucial reprogramming factors, this strategy may also suffer from biased identification of factors that efficiently activate the *α*MHC promoter, while excluding those that may promote full reprogramming. To address this issue, Protze et al. [27] screened 120 different triplet combinations of 10 candidate reprogramming factors for their ability to activate expression of five cardiac genes (Myh5, Myl2, Actc1, Nkx2.5, and Scn5a) in mouse fibroblasts. Expression of Tbx5, Mef2c, and Myocd together upregulated the most cardiac genes examined. Although fibroblasts transduced with these three factors indeed expressed cardiac cell fate indicators, such as contractile proteins, cardiac-like sodium and potassium currents, and inducible action potentials, these iCMs failed to spontaneously beat. Other groups developed alternative criteria for reprogramming factor combination screening. Christoforou et al. [28] assayed different combinations of 10 transcription factors for their ability to induce cardiomyocyte conversion in MEFs. Criteria for identifying factor combinations included activation of cardiac-specific promoters and specific cardiac genes, cardiac morphologic features, and functional capacities including resting membrane potential and spontaneous beating. Through this method, the authors determined that Myocd and SRF could improve GMT reprogramming; however, no spontaneous contractile activity was observed.

In another recent study, Addis et al. [29] sought to optimize direct cardiac conversion by using a quantifiable calcium-reporter, GCaMP driven by the cTnT promoter, to screen candidate factor combinations for the ability to induce functional transdifferentiation in MEFs. Using this method Hand2, Nkx2.5, Gata4, Mef2c, and Tbx5 (HNGMT) were identified as the optimal combination for inducing robust calcium oscillation and an increase in spontaneously beating iCMs compared to GMT reprogramming alone. In a subsequent study utilizing the same calcium reporter, Ifkovits et al. [30] showed that the small molecule TGF-*β* Inhibitor SB432542 increased the efficiency of HNGMT reprogramming 5-fold.

While most of these studies focused on transcription-factor mediated reprogramming, Jayawardena et al. [5] reported successful conversion of neonatal mouse fibroblasts to cardiomyocyte-like cells* in vitro *through the combination of 4 miRs: miR-1, -133, -208, and -499. Addition of a JAK inhibitor (JI1) resulted in enhanced reprogramming efficiency as well as spontaneously beating iCMs. Extending the capabilities of the miR reprogramming, Muraoka et al. [31] showed that addition of miR-133a to the combination of GMT in MEFs yielded a 7-fold increase in beating iCMs compared with GMT reprogramming alone; miR-133a was shown to repress Snai1, a master regulator of epithelial-to-mesenchymal transitions, suggesting that suppressing fibroblast signatures may be critical in reprogramming fibroblasts to iCMs. Taken together, these studies demonstrate that many approaches and criteria are used to screen for cardiac reprogramming factors and to validate cardiac cell fate conversion. For instance, some studies evaluate expression of cardiac specific genes by targeted qPCR, which is not as comprehensive an evaluation as global transcriptome profiling. Regardless, the numerous recent developments in direct cardiac reprogramming* in vitro* (Figure 1) are promising for significant advances in current methods and understanding of this emerging technology.

## 5. *In Vivo* Direct Cardiac Reprogramming

Building upon decades of cardiovascular biology research, which elucidated transcriptional factor networks important in embryonic heart development, in 2009 Takeuchi and Bruneau [32] discovered that overexpression of transcription factors of Gata4, Tbx5, and chromatin remodeling protein, Baf60c, in noncardiogenic mesoderm was sufficient to induce spontaneously contracting cardiomyocytes in 50% of transfected mouse embryos. In the absence of Tbx5 from this combination of reprogramming factors, differentiation into the cardiac lineage was still promoted; however, resultant cardiomyocytes did not spontaneously contract. The demonstration that robust beating iCMs could be induced* in vivo* with coexpression of Gata4, Tbx5, and Baf60, which together were not identified as promoting* in vitro* cardiac reprogramming [4, 32] suggests that elements of the native microenvironment may be conducive to the reprogramming process. Direct cardiac reprogramming* in vivo* might, therefore, be expected to yield significantly higher conversion efficiencies than* in vitro.*

To test this hypothesis, Qian et al. [3] retrovirally delivered GMT to peri-infarct areas in 2-month-old male mice after coronary artery ligation and observed an increased efficiency of iCM conversion compared to* in vitro* GMT reprogramming. Cre-based lineage tracing studies were used to determine the origin of retrovirally infected iCMs. In the reprogrammed infarct area, a portion of cells with sarcomere structures and cardiomyocyte morphology were positive for both fibroblast and cardiomyocyte lineage markers (Fsp1-Cre and Periostin-Cre). Importantly, lineage tracing using *α*MHC-MerCreMer mice was used to rule out the possibility that cell-cell fusion was responsible for observed iCMs. These* in vivo* iCMs shared many features with endogenous cardiomyocytes. They were binucleate, with clearly assembled sarcomeres, and they demonstrated cardiac-specific gene expression. Furthermore, single-cell analysis of iCM electrophysiology revealed that half of iCMs exhibited ventricular cardiomyocyte-like action potentials upon electric stimulation. Remarkably, iCMs electrically coupled with endogenous iCMs, which is crucial for potential future clinical applications. Importantly, GMT delivery* in vivo* to infarcted mouse myocardium also showed decreased scar formation and improved cardiac functioning, as assessed by MRI and echocardiography.

Along with this study on* in vivo* cardiac reprogramming, other independent laboratories have reported modifications to GMT factor reprogramming methods. Song et al. [24] found that addition of Hand2 to the GMT factors could efficiently induce expression of cardiac markers* in vivo*. Also, the resultant iCMs were similar to endogenous cardiomyocytes in morphology, with rod shaped structures and assembled sarcomeres, as well as in functionality, with many iCMs generating Ca^2+^ transients and action potentials. As with GMT factor reprogramming, the GHMT factors were capable of decreasing scar size and improving pump functioning in infarcted mouse hearts.

In another example of* in vivo* GMT factor reprogramming, Inagawa et al. [33] demonstrated similar findings to Qian et al. [3]. However, the novelty of this study involved alternative experimental methods aimed at improving reprogramming efficiency. In contrast to the multiple retroviral deliveries of GMT factors used by Qian et al. [3], Inagawa et al. [33] used a single promoter polycistronic retrovirus containing GMT with intervening sequences of 2A self-cleaving peptides, which permitted more homogenous GMT expression. Additionally, immunosuppression of mice treated with retrovirus aided in preventing immune-mediated destruction of transduced cells. Although this study lacked functional characterization of iCMs, the novel experimental modifications significantly improved* in vivo* iCM generation. Building on the goal of more homogenous gene expression of reprogramming factors, Mathison et al. [34] also generated a single-promoter polycistronic vector encoding GMT and 2A self-cleaving peptides. Compared to multiple viral delivery of GMT, polycistronic GMT delivery to infarcted rat hearts resulted in a 2-fold increase in iCM generation as well as enhanced ventricular function.

Adding another twist to transcription-factor-mediated cardiac reprogramming, several other groups began investigating the impact of small molecule addition to the GMT cocktail of factors. Mathison et al. [35] added expression of three major VEGF isoforms to GMT factor expression in infarcted rat hearts. Compared to treatment with GMT alone, treatment with GMT factors and VEGF in infarcted rat hearts resulted in decreased fibrosis and a 4-fold increase in ejection fraction. Supporting the role of additional compounds in enhancing GMT factor reprogramming, Srivastava et al. [36] demonstrated that addition of a fibroblast activator, thymosin *β*4, to the GMT reprogramming cocktail also increased the number of reprogrammed iCMs. Alternative methods to transcription factor reprogramming have also been demonstrated. Jayawardena et al. [5] reprogrammed mouse fibroblasts to cardiomyocyte-like cells* in vivo* using the combination of miR-1, -133, -208, and -499. Later, it was shown that JAK inhibitor with miR-1 was alone sufficient to reprogram fibroblasts into cardiomyocyte-like cells. More recently, Jayawardena et al. [37] showed that delivery of this miR combination to peri-infarct regions of mouse myocardium resulted in cardiomyocyte-like cells with mature features including sarcomere structure, excitation-contraction coupling, and action potentials similar to those of mature ventricular cardiomyocytes. Serial echocardiography also revealed progressive improvements in left ventricular functioning starting 1 to 2 months postsurgery and enhanced functioning at 3-month follow-up. Together these novel approaches to* in vivo* cardiac reprogramming show significantly greater reprogramming efficiencies than* in vitro* experiments and demonstrate the crucial role of the microenvironment in nurturing iCM generation. Local paracrine agents, tensile forces, extracellular matrix, and other unknown factors likely facilitate maturation of iCMs, and surely this will be an active area of exciting new developments to come.

## 6. Direct Reprogramming of Human Fibroblasts to a Cardiac Cell Fate

Considering the abundant supply of cardiac fibroblasts (>50%) in the human heart [38], a clinically important step for the direct cardiac reprogramming field will be to translate reprogramming technology to human fibroblasts. Nam et al. [6] reported successful induction of cTnT expression in human fibroblasts by use of transcription factors Gata4, Hand2, Tbx5, and Myocd together with miR-1 and -133. Although iCMs showed sarcomere assembly, expression of some cardiac genes, downregulation of fibroblast genes, and Ca^2+^ transients, the vast majority of iCMs failed to upregulate a host of cardiac genes and spontaneously beating iCMs were sparse, suggesting a partially reprogrammed state in the majority of transduced cells. Using only transcription factors, Fu et al. [7] aimed to demonstrate* in vitro* reprogramming of human fibroblasts to iCMs with the GMT factors. GMT alone did not result in reprogramming of human fibroblasts; however, addition of MESP1 and ESRRG to the GMT factor mix resulted in global shifts in cardiac gene-expression and changes in phenotype toward cardiac morphology [7]. Adding Myocd and ZFPM2 to this cocktail further improved reprogramming, with resultant iCMs assembling sarcomeres and exhibiting cardiac cell-like electrophysiology [7]. However, the iCMs were not observed to beat, even long after reprogramming, despite demonstrating similar global gene expression levels as were achieved by GMT reprogramming of mouse fibroblasts* in vitro* [7]. Wada et al. [8] also demonstrated that GMT factors, MESP1 and Myocd together were capable of inducing cardiac gene expression in human fibroblasts, although the iCMs did not beat spontaneously. Interestingly, culturing these iCMs with mouse cardiomyocytes resulted in their beating [8]. While these studies demonstrate that human fibroblasts are indeed amenable to cardiac reprogramming, they also underscore the need to better understand the barriers to complete reprogramming as well as the mechanism of reprogramming. Recently, several groups have made exciting headway in these directions.

## 7. Challenges and Future Objectives

While many exciting developments have been made in the emerging field of direct cardiac reprogramming, many challenges must be addressed to move this technology closer toward applications in disease modeling, drug screening and ultimately toward treating human heart diseases. One challenge is the current inefficiency of* in vitro* cardiac reprogramming in both human and mouse fibroblasts. The first reported method of* in vitro* reprogramming fibroblasts into iCMs by GMT cocktail demonstrated an efficiency of iCM generation at <1%, though the efficiency was remarkably higher* in vivo* [3, 4]. Later, the use of a single-promoter, polycistronic vector encoding the GMT factors increased the number of iCMs generated* in vivo* compared to use of separate virus delivery of GMT [33, 34]. Most recently, Wang et al. [39] demonstrated that an important determinant in both the efficiency and the quality of iCM generation relies on stoichiometric expression of the GMT factors [39]. By testing the reprogramming efficiencies of 6 polycistronic constructs encompassing all possible combinations of the GMT factors, Wang et al. [39] determined that high expression of Mef2c, which was observed with the polycistronic MGT construct, resulted in a 10-fold increase in the number of beating iCMs compared to separate viral transduction of the GMT factors. Together these results point to homogenous and stoichiometric expression of reprogramming factors as key elements of successful reprogramming. While many studies have achieved successful reprogramming, other groups have had less success [40], which further emphasizes the requirement for optimization of experimental conditions. Nevertheless, continued progress in increasing the efficiency of* in vitro* reprogramming will be necessary to facilitate future studies into the mechanism of reprogramming.

Importantly, it is also necessary to understand how epigenetic barriers are overcome and how new epigenetic states are established and maintained during reprogramming into functionally mature iCMs. Epigenetic barriers may in part account for cell-to-cell differences in amenability to complete reprogramming and thus explain why some populations of iCMs remain partially reprogrammed or appear refractory to complete reprogramming, while others are reprogrammed to functionally mature states. Given the increased efficiency of* in vivo* reprogramming [3], it is likely that small molecules, paracrine factors, and other unknown factors and mechanisms inherent to the native cardiac environment play a role in the functional maturation of iCMs. Identifying these maturation-promoting factors as well as elucidating the epigenetic and transcriptional changes in cells undergoing reprogramming will be important for the development of iCMs for* in vitro* applications, such as drug toxicity screening and disease modeling, or* in vivo* applications, such as* in situ* generation of iCMs following myocardial infarction.

Given the high demand for regenerative therapies to treat injuries in essential, poorly regenerative organs, such as the heart, a direct cellular reprogramming approach may be especially well suited for treatment of cardiac disease. Currently, regenerative cardiac therapies undergoing clinical trials involve explant culture of autologous cardiac-derived cells (CDCs) and transplant in patients with chronic heart failure or several months after MI [41]. Transplant-based CDC therapies, such as autologous human cardiac-derived stem cells, have shown some functional benefit and reduced infarct scar size; however, evidence shows lack of significant long-term cell engraftment, though tissue-engineering approaches may improve engraftment. Also, the requirement for several months of explant culture to generate cardiomyocytes for individual patients hinders future scaling-up of this treatment as well as utilization in acute MI settings. Given that direct cardiac reprogramming can generate iCMs* in situ *in peri-infarct regions following acute coronary events in animal models, this nascent technology could potentially address issues faced by other cardiac regenerative approaches. Direct cardiac reprogramming is, therefore, an area of study that merits continued investigation. Despite the many challenges in this emerging field of study, significant progress is being made to advance this technology toward regenerative medicine applications in the near future.
